# Changes in ambient temperature increase hospital outpatient visits for allergic rhinitis in Xinxiang, China

**DOI:** 10.1186/s12889-021-10671-6

**Published:** 2021-03-27

**Authors:** Jianhui Gao, Mengxue Lu, Yinzhen Sun, Jingyao Wang, Zhen An, Yue Liu, Juan Li, Zheng Jia, Weidong Wu, Jie Song

**Affiliations:** 1 Xinxiang Medical University, Xinxiang, 453003 China; 2 Henan International Collaborative Laboratory for Health Effects and Intervention of Air Pollution, Xinxiang, 453003 China; 3 National Institute of Environmental Health, Chinese Center for Disease Control and Prevention, Beijing, 100021 China; 4 Xinxiang Central Hospital, Xinxiang, 453001 China

**Keywords:** Ambient temperature, Allergic rhinitis, Outpatient, Distributed lag non-linear model

## Abstract

**Background:**

The effect of ambient temperature on allergic rhinitis (AR) remains unclear. Accordingly, this study aimed to explore the relationship between ambient temperature and the risk of AR outpatients in Xinxiang, China.

**Method:**

Daily data of outpatients for AR, meteorological conditions, and ambient air pollution in Xinxiang, China were collected from 2015 to 2018. The lag-exposure-response relationship between daily mean temperature and the number of hospital outpatient visits for AR was analyzed by distributed lag non-linear model (DLNM). Humidity, long-time trends, day of the week, public holidays, and air pollutants including sulfur dioxide (SO_2_), and nitrogen dioxide (NO_2_) were controlled as covariates simultaneously.

**Results:**

A total of 14,965 AR outpatient records were collected. The relationship between ambient temperature and AR outpatients was generally M-shaped. There was a higher risk of AR outpatient when the temperature was 1.6–9.3 °C, at a lag of 0–7 days. Additionally, the positive association became significant when the temperature rose to 23.5–28.5 °C, at lag 0–3 days. The effects were strongest at the 25th (7 °C) percentile, at lag of 0–7 days (RR: 1.32, 95% confidence intervals (CI): 1.05–1.67), and at the 75th (25 °C) percentile at a lag of 0–3 days (RR: 1.15, 95% CI: 1.02–1.29), respectively. Furthermore, men were more sensitive to temperature changes than women, and the younger groups appeared to be more influenced.

**Conclusions:**

Both mild cold and mild hot temperatures may significantly increase the risk of AR outpatients in Xinxiang, China. These findings could have important public health implications for the occurrence and prevention of AR*.*

**Supplementary Information:**

The online version contains supplementary material available at 10.1186/s12889-021-10671-6.

## Introduction

Allergic rhinitis (AR) is triggered by a spectrum of environmental allergens and is considered an immunoglobulin E (IgE) mediated type 1 hypersensitivity illnesses [1]. AR is typically classified according to the four common nasal symptoms: sneezing, itching, rhinorrhea, and nasal congestion. In addition, AR may result in itchy upper jaw, coughing, and swollen watery eyes [2]. Furthermore, AR may impair cognitive function and sleep quality, causing fatigue and irritability. Other negative health conditions concluding hypertension, sinusitis, asthma, and otitis media are also commonly caused by AR [3, 4]. Therefore, AR can significantly affect patient quality of life, and contribute to a decline in school performance, work productivity, and social life [3, 5], especially during the peak pollen season [1]. AR morbidity has soared worldwide in recent decades [6]. Previous studies have demonstrated that the prevalence of AR varies between different countries. However, more than 20% of the population is affected by AR worldwide [7]. In some countries, more than 50% of citizens were reported to have AR [8–10]. Estimates showed that the medical costs associated with loss of work efficiency caused by AR was US$ 1664 per patient, per year [11]. Consequently, the overall medical and economic burdens associated with AR are enormous.

Increasing evidence have revealed that both environmental and genetic factors play important roles in the etiology of allergic diseases, including AR [4, 12, 13]. Ambient temperature is a major environmental factor, and reports have frequently related it to health outcomes with significant lag effects such as global overall mortality, respiratory diseases, cardiovascular diseases, and cerebrovascular diseases [14–18]. Moreover, several studies have documented that high temperatures were related to respiratory problems [19]. However, the evidence for this relationship is scarce and inconsistent. Population studies in the Pacific Rim showed that the AR prevalence was positively correlated with mild hoter temperatures [20], whereas in the northern hemisphere, individuals with AR often had increased respiratory symptoms due to frequent cold air exposure [21]. Recent evidence suggests that cold weather may aggravate symptoms, especially in patients with certain underlying respiratory diseases [22]. A high incidence of respiratory symptoms was also reported in cold indoor environments [23]. Another population-based study demonstrated that in adults with certain respiratory illnesses, increased symptoms occurred in cold environments and were more pronounced in patients with rhinitis [24].

The development of climate change and global warming due to increased atmospheric carbon dioxide (CO_2_) levels has been widely accepted over the past decades. Due to its very large population, even a minor temperature increase could potentially cause huge health losses in China. However, the potential effect of ambient temperatures on AR has not been directly examined in the Chinese population. Consequently, we performed a time-series study to explore the lag-exposure-response relationship between the prevalence of AR-related outpatient visits and ambient temperature.

## Methods

The population of Xinxiang, China (35.18′N, 113.52′E; area 8269 km^2^) was approximately 5.67 million at the end of 2014. The city is located in north of Henan province, on the north side of the Yellow River. Our research area was limited to the four traditional urban districts (422 km^2^). Xinxiang has a typical temperate continental monsoon climate, with prevailing northeasterly winds.

### Hospital outpatient data

Six hospitals are distributed throughout the urban districts of Xinxiang, and hospital-specific geographic information was described in our previous research [25]. The study excluded one military hospital and hospital lacking an electronic information system from the study. The four remaining hospitals (Xinxiang Central Hospital, Xinxiang First People’s Hospital, Xinxiang Second People’s Hospital, and the Third Affiliated Hospital of Xinxiang Medical University) were included. Computerized records of AR-related visits from January 1, 2015 to December 31, 2018 were retrieved from the health information system of each hospital. Subsequently, the data were cleaned and quality controlled as described in our previous study [26]. The number of daily AR visits was summarized based on the J30 code as determined by the International Classification of Diseases (10th revision) for AR.

### Ambient temperature and air pollutants data

Daily meteorological parameters, including relative humidity, maximum, minimum, and mean daily temperatures in Xinxiang from January 1, 2015 to December 31, 2018 were collected from the China Meteorological Data Sharing Service System (http//106.37.208.235.20035/). Additionally, daily mean concentrations of nitrogen dioxide (NO_2_) and Sulfur dioxide (SO_2_) pollutants were measured at four fixed-site stations administrated by the Xinxiang Ministry of Ecology and Environment.

### Statistical analysis

Due to lag effects and the non-linearity of correlations between temperature and specific diseases reported in previous studies [27–30], a distributed lag non-linear model (DLNM) was applied in this study to examine the association between AR outpatient visits and ambient temperature [31]. The DLNM models combines prediction and lag effect in a bi-dimensional matrix (so called cross-basis), and is effective for estimating prediction across a possible maximum lag period.

A generalized additive model was adopted to link the outcome (daily AR outpatient visits) and exposure (daily mean ambient temperature). Additionally, several covariates were incorporated to control for potential confounding effects as previously described [32]. Covariates included (1) unmeasured long-term and seasonal trends in AR incidence, controlled by a natural cubic smooth function with a degree of freedom (df) of 7 per year; (2) relative humidity; (3) day of the week; (4) holidays, and (5) daily mean concentrations of NO_2_ and SO_2_ (previously reported as potential hazard factors for AR) [25]. To optimize the model, we calculated the Akaike information criterion (AIC) value and selected the minimal structure as the best model. And in order to check the stability of our model, we selected alternative df for time space with 4–10 per year.

The 1st (− 2 °C), 25th (7 °C), 75th (25 °C), and 99th (32 °C) daily mean temperature percentiles relative to the median temperature were calculated for the relative risks (RR) and 95% confidence intervals (CIs) of the AR outpatient visits at cumulative lag effects from 0 to 28 days, respectively. These measurements were applied to analyze the effects of cold, mild cold, mild hot, and hot temperatures on AR outpatient visits. Moreover, we conducted sex and age (< 15 years, 15–64 years, and ≥ 65 years) group stratification analyses to determine their effects on the risk of AR outpatient visits. The statistical significance of differences between effect estimates of the strata by calculating the 95% confidence intervals (CI) as $\left({\hat{Q}}_1-{\hat{Q}}_2\right)\pm 1.96\sqrt{{\left(\mathrm{S}{\hat{\mathrm{E}}}_1\right)}^2+{\left(\mathrm{S}{\hat{\mathrm{E}}}_2\right)}^2}$, where ${\hat{Q}}_1$ and ${\hat{Q}}_2$ are the estimates for two categories, and $\mathrm{S}{\hat{\mathrm{E}}}_1$ and $\mathrm{S}{\hat{\mathrm{E}}}_2$ are their respective standard errors [33].

All statistical analyses were completed by DLNM and MGCV packages in R software (Version 3.3.3). A *p* value of < 0.05 were considered statistically significant.

## Results

Between January 1, 2015 and December 31, 2018, there were 14,965 hospital outpatient visits due to AR in Xinxiang, China. A total of 7912 male and 7050 female outpatients (3 records missing) were collected (Table 1). Of the total AR outpatients, 2159 were less than 15 years of age, 11,945 were 15–64 years, and 861 were at least 65 years of age. The annual mean temperature during this time period was 16 °C, and the temperature ranged from − 6.1 °C to 34.6 °C. The annual mean concentrations of NO_2_ and SO_2_ were 48.0 μg/m^3^ and 33.0 μg/m^3^, respectively.

**Table 1:** Daily hospital outpatients for allergic rhinitis, meteorological and air pollution factors in Xinxiang, 2015–2018

No. of daily outpatients for allergic rhinitis (J30)	Mean (SD)	P1	P25	P50	P75	P99
	11 (8)	0	5	8	13	40
Sex						
Female	5 (5)	0	2	4	7	19
Male	6 (5)	0	2	4	7	23
Age						
<15 years	2 (2)	0	0	1	2	9
15–64 years	9 (7)	0	4	7	10	33
≥ 65 years	1 (1)	0	0	0	1	3
Meteorological measures						
Mean temperature (°C)	16 (10)	−2	7	17	25	32
Humidity (%)	60 (17)	22	48	62	74	93
Air pollutant concentration (μg/m3)						
NO_2_	48 (21)	16	32	45	61	111
SO_2_	33 (25)	6	16	26	42	122

^*NO*^_*2*_^Nitrogen Dioxide, *SO*^_*2*_^Sulfur Dioxide, *P1* 1st percentile, *P25* 25th percentile, *P50* 50th percentile, *P75* 75th percentile, *P99* 99th percentile;^

Table 2 presents the estimated RRs of daily mean temperatures on AR outpatient visits, adjusted for confounding factors, including long-time trends, day of the week, relative humidity, and ambient concentrations of NO_2_ and SO_2_ at the 1st, 25th, 75th, and 99th temperature percentiles relative to the median temperature (17 °C) over different lag times. Non-significant associations between cold temperatures (− 2 °C, 1st percentile) and AR outpatient visits were observed for all lag times. The heat temperature (32 °C, 99th percentile) had a negative effect on AR outpatient at a lag of 0–14 days, 0–21 days, and 0–28 days. The results show that either mild cold (7 °C, 25th percentile) or mild hot (25 °C, 75th percentile) temperatures could increase the risk for AR. Of note, mild cold temperatures had slightly longer lag times (lag 0–6 days, lag 0–7 days) and the mild hot temperatures had slightly shorter lag times (lag 0–2 days, lag 0–3 days).

**Table 2:** Relative risks of allergic rhinitis associated with daily mean temperature among selected cutoff points

Lag effects (days)	1st percentile relative to median temperature RR (95% CI)	25th percentile relative to median temperature RR (95% CI)	75th percentile relative to median temperature RR (95% CI)	99th percentile relative to median temperature RR (95% CI)
Lag 0	0.98 (0.75–1.27)	1.03 (0.89–1.18)	1.00 (0.91–1.11)	0.85 (0.71–1.02)
Lag 0–1	0.95 (0.72–1.26)	1.02 (0.87–1.18)	1.09 (0.98–1.21)	0.96 (0.79–1.18)
Lag 0–2	0.98 (0.73–1.33)	1.03 (0.87–1.21)	1.14 (1.01–1.27)*	1.05 (0.85–1.31)
Lag 0–3	1.04 (0.75–1.44)	1.09 (0.91–1.30)	1.15 (1.02–1.29)*	1.09 (0.86–1.37)
Lag 0–4	1.07 (0.76–1.51)	1.16 (0.96–1.41)	1.13 (0.99–1.29)	1.10 (0.86–1.40)
Lag 0–5	1.08 (0.75–1.55)	1.23 (1.00–1.51)	1.10 (0.96–1.27)	1.08 (0.83–1.40)
Lag 0–6	1.08 (0.73–1.59)	1.27 (1.02–1.58)*	1.07 (0.92–1.23)	1.05 (0.80–1.38)
Lag 0–7	1.11 (0.73–1.68)	1.32 (1.05–1.67)*	1.05 (0.90–1.22)	1.02 (0.77–1.37)
Lag 0–14	0.80 (0.44–1.45)	1.26 (0.90–1.77)	0.91 (0.73–1.12)	0.59 (0.39–0.88)*
Lag 0–21	1.02 (0.47–2.20)	1.50 (0.98–2.30)	0.79 (0.59–1.04)	0.28 (0.16–0.47)*
Lag 0–28	1.46 (0.54–3.96)	1.61 (0.91–2.82)	0.79 (0.54–1/16)	0.24 (0.12–0.50)*

*p<0.05. *RR* relative risk

**Fig. 1:**
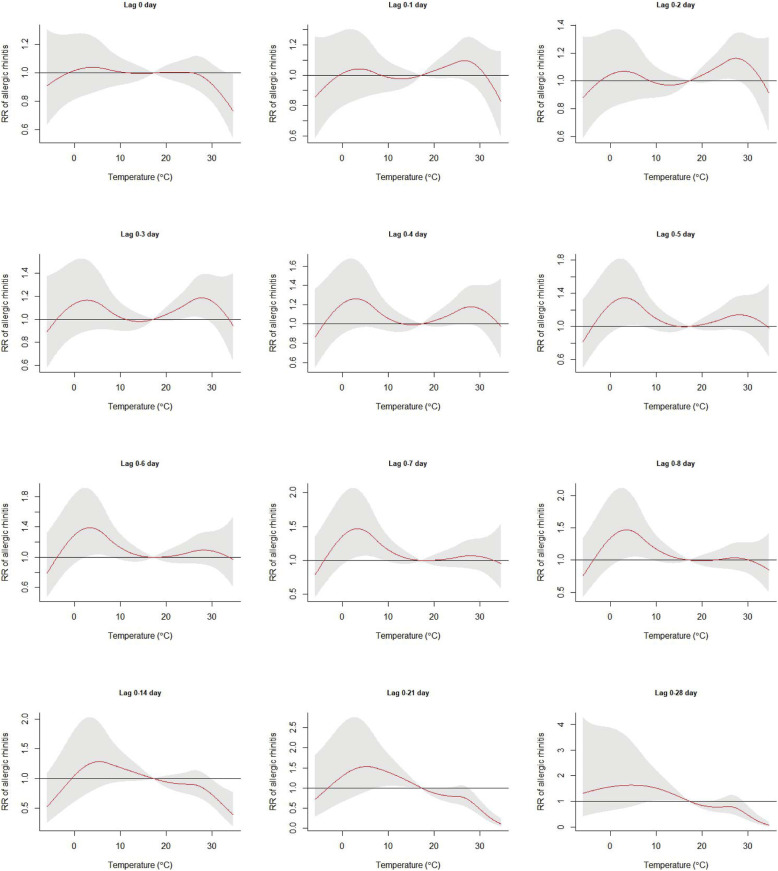
Exposure-response curves of daily mean temperature and cumulative relative risk of daily allergic rhinitis (reference temperature at 17 °C) at different lag structures

Figure 1 depicts the lag pattern of the temperature’s effect on AR using a series lag structures. Mild cold temperatures from 1.6 °C to 9.3 °C may increase AR prevalence at a lag of 0–7 days (Fig. 1), and mild hot temperatures between 23.5 °C and 28.5 °C may increase the AR risk at a lag of 0–3 days (Fig. 1).

**Fig. 2:**
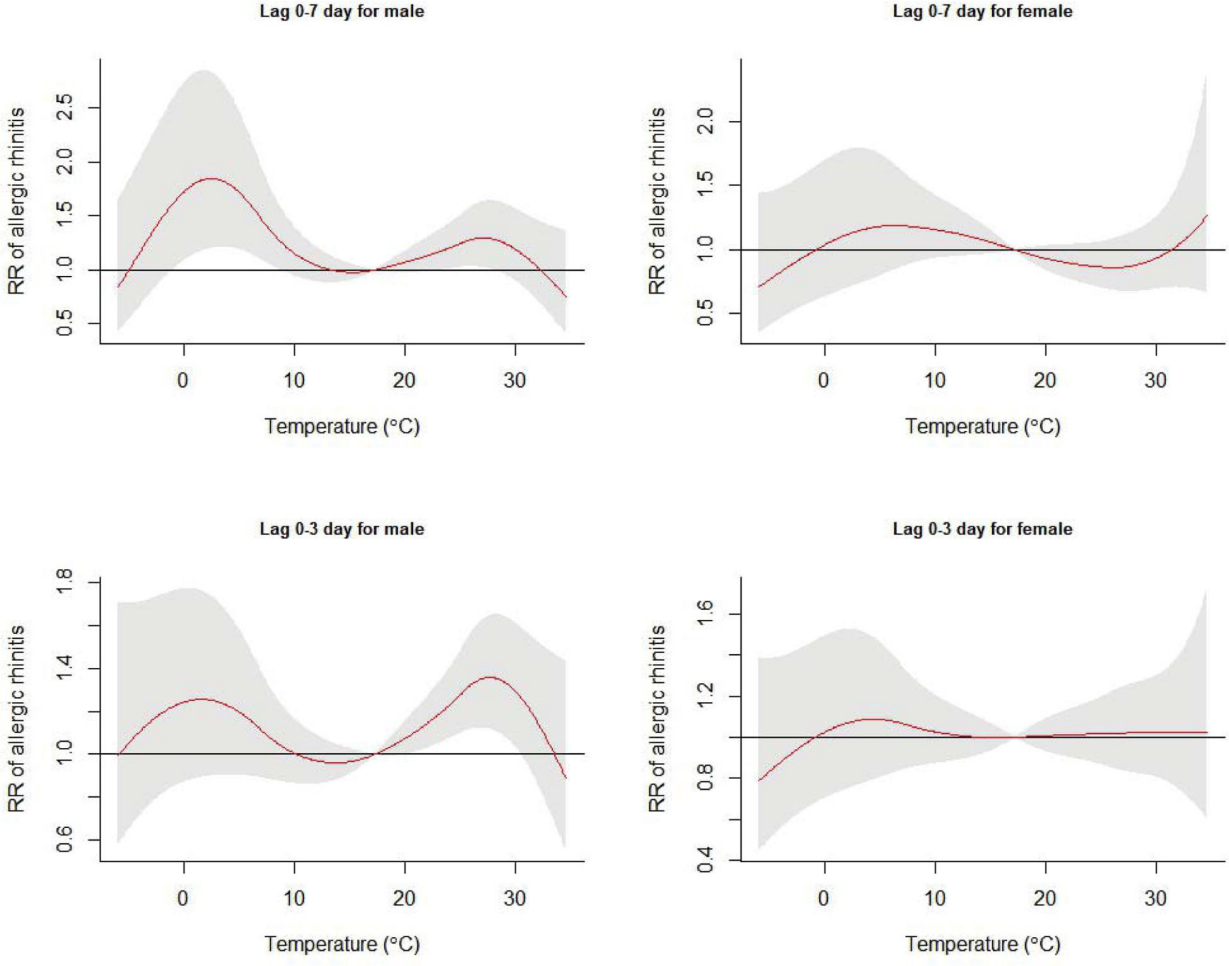
Exposure-response curves of daily mean temperature and cumulative relative risk of daily allergic rhinitis (reference temperature at 17 °C) at a lag of 0–3 days and a lag of 0–7 days, stratified by sex

**Fig. 3:**
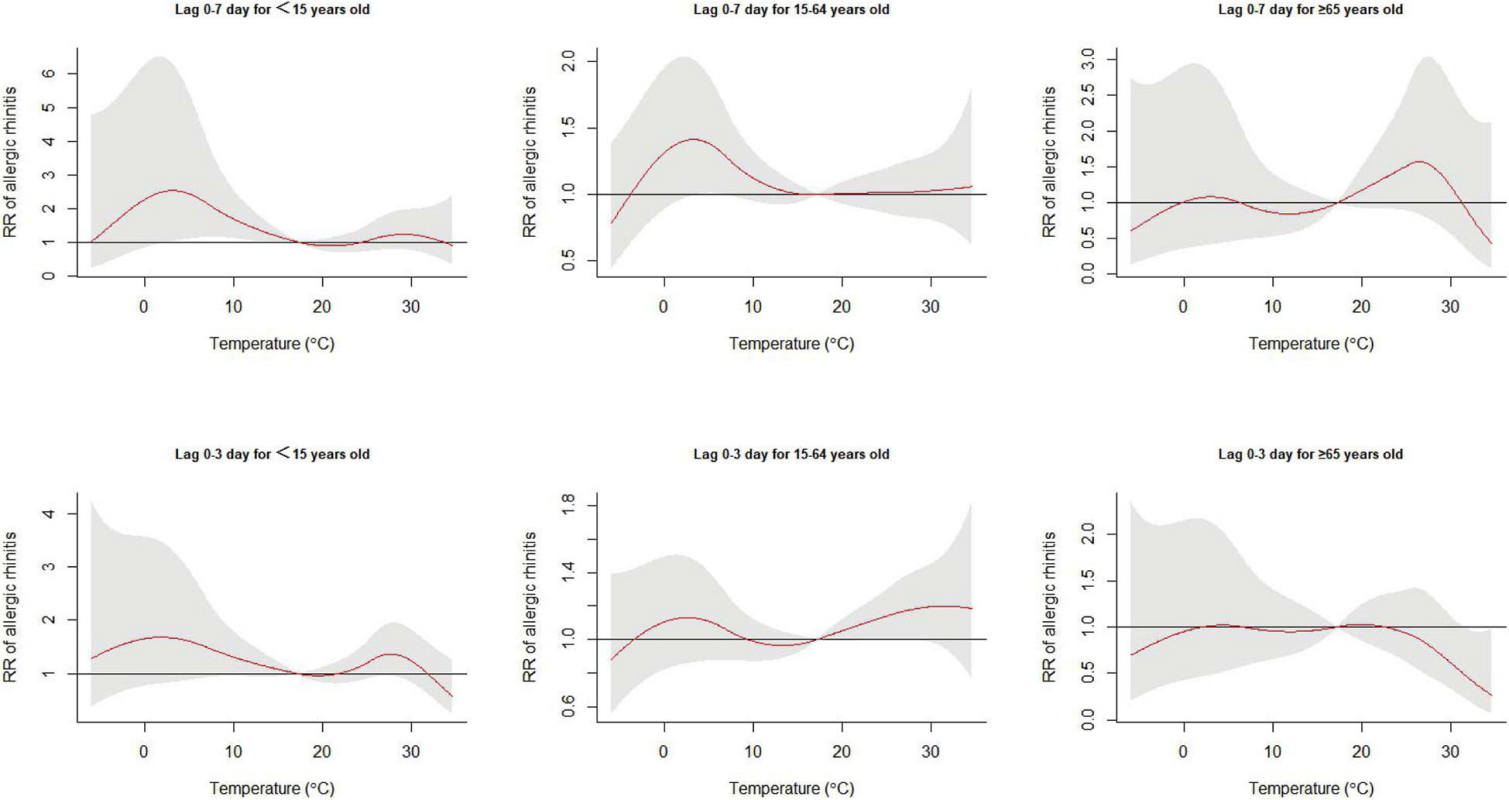
Exposure-response curves of daily mean temperature and cumulative relative risk of daily allergic rhinitis (reference temperature at 17 °C) at a lag of 0–3 days and a lag of 0–7 days, stratified by age

The exposure-response curves of daily mean temperature on AR hospital outpatients at a lag of 0–7 and 0–3 days stratified by sex are presented in Fig. 2. The results showed that men were more susceptible than women to temperature, including mild cold temperatures at a lag of 0–7 days, and mild hot temperatures at a lag of 0–3 days. The age-stratified results showed that the young group (< 15 years of age) was sensitive to mild cold temperatures at a lag of 0–7 days (Fig. 3).

The sensitivity analysis results proved that the acute effects did not change substantially with the adjustment of smoothness of time using alternative df from 4 to 10 per year (Supplement Fig. 1). That suggesting our models are robust and probably not attributable to chance.

## Discussion

In this study, we observed that both mild cold and mild hot temperatures were significantly associated with an increased risk of AR in Xinxiang, China. An “M”-shaped exposure-response curve for AR and daily temperature was observed. Males and the younger group (< 15 years of age) may be more sensitive to ambient temperature. To our knowledge, this was one of the few studies to directly investigate the relationship between ambient temperature and AR outpatients among Asian population.

Many studies have found positive associations between hot temperatures and AR risk [34–37]. Similarly, reports showed that high temperatures were also associated with respiratory problems [19, 38]. Zhang et al. found that increased levels of SO_2_, NO_2_, and PM_10_ increased a higher AR risk in the warm season than that in the cold season [39]. However, another study conducted in Finland indicated that respiratory symptoms including asthma and AR were increased in cold weather [34]. In this study, we found a significant positive association between AR and mild cold or mild hot temperatures, however, we did not find a significant association between AR and hot or cold temperatures. This may be due to behavioral and environmental changes in summer and winter, such as fewer outdoor activities and the use of air conditioners or central heating systems, reducing exposure to extreme temperatures. The differences between our current results and previous findings may be partially explained by differences in the region, climate, and temperature tolerance associated with the local population. Previous studies have found that populations at middle and low latitudes may adapt to high temperatures, and populations at high latitudes may adapt to low temperatures [40].

Other studies have demonstrated that ambient temperature can alter vegetation patterns spatially and temporally, and is associated with allergic symptoms in several ways [37, 41–43]. Pollen is a primary risk factor for AR. A study by Beggs indicated that changes in climate including increases in greenhouse gases (CO_2_), may affect aeroallergens through plant distribution, pollen quantity, and season [44]. Evidence suggests that pollen grown at higher temperatures possesses significantly stronger allergenicity [45]. In addition, ambient temperature may also increase the risk of allergies by affecting the immune system since high temperature may promote increased levels of neutrophils [46, 47]. Neutrophils can release mediators, leading to bronchial epithelial damage and airway obstruction [46].

Another noteworthy finding from our study is the significant relationship between AR outpatient visits and mild cold temperatures at a lag of 0–7 days, and also between AR visits and mild hot temperatures at a lag of 0–3 days. One study conducted in Guangzhou, China, also found that the effects of warmer temperatures appeared immediately, whereas the effects of colder temperatures were delayed but persisted longer [48]. Warmer temperatures stimulate thermoregulation to maintain heat balance in the human body. During this process, increased heat dissipation is facilitated by accelerating blood circulation and increased sweating. In turn, these changes increase dehydration and salt loss, leading to increased blood viscosity and cholesterol levels, which can result in acute diseases attacks [49]. Warmer temperatures also stimulate plant growth, leading to more environmental allergens such as pollen, increasing the incidence of AR.

Stronger temperature effects on AR outpatient visits were observed in males than in females, at both mild hot and mild cold temperatures. One possible explanation is that males may have participated in more outdoor work resulting in greater exposure to higher temperatures. However, other studies have shown that females are more susceptible to temperature-related health hazards [50, 51]. Clearly, further studies are required to investigate the specific underlying mechanisms for these effects.

Previous studies have observed that adverse effects of temperature on older adults and children are often higher than average [52]. Our study also observed a higher estimate for AR risk in the younger and older groups, however, the effects in older adults were non-significant. It is noteworthy that the daily outpatient number of the younger and older groups were very small, this might cause some instability to the result. Further studies are needed to elucidate reasons for this discrepancy.

Our findings will contribute to understanding the influence of ambient temperature on AR, and to predict the impact of increased average temperatures due to climate change on the health of individuals with allergies. In addition, as global average temperatures and the incidence of allergies continue to increase, our results will provide a basis for future studies on the effects of increased temperature specifically on AR, and may also aid in the formulation of relevant health protection strategies.

Nevertheless, there are some limitations in our study. First, ecological bias was inevitable. For instance, outdoor fixed-site monitoring data were used as surrogates for individual temperature exposure levels, which may have caused measurement errors since individuals stay indoors most of the time [53]. Although the magnitude of the error is difficult to quantify, in general it would tend to bias the risk estimates downwards [54]. Second, individual lifestyles (such as the use of air conditioning and heating systems) would significantly alter the exposure temperature for a proportion of the population. Third, concentrations of several allergens related to AR (such as plant pollen) are significantly associated with temperature [55], and their spatial distribution may also differ significantly. Together, these factors may affect the magnitude of the calculated estimates. Fourth, daily outpatient number for the younger (< 15 years) and the old (≥65 years) were small, their results might be unrobust. Finally, this study gathered data from only one city, and therefore the general applicability of the results may be limited.

## Conclusions

In summary, mild cold and mild hot temperatures both significantly increased the AR hospital outpatient visits in Xinxiang, China. This adverse effect was especially pronounced in males and in the younger population (< 15 years of age). As the global average temperature increases, individuals with AR or other allergies may develop heat-related health problems. Our study may have important public health implications for controlling and preventing adverse health effects associated with exposure to increased air temperature.

## Supplementary Information

**Additional file 1: Figure S1.** Relative risks of allergic rhinitis associated with daily mean temperature at 75th percentile relative to median temperature at lag02 day using different degrees of freedom per year.

## Data Availability

The datasets used and analyzed during the current study available from the corresponding author on reasonable request.
