# Supplementary material for: Changes in ambient temperature increase hospital outpatient visits for allergic rhinitis in Xinxiang, China
Source: BMC Public Health. 2021 Mar 27;21:600. doi: 10.1186/s12889-021-10671-6 (PMC8004401; doi:10.1186/s12889-021-10671-6)
Supplement: Supplementary file 1 — Additional file 1: Figure S1. Relative risks of allergic rhinitis associated with daily mean temperature at 75th percentile relative to median temperature at lag02 day using different degrees of freedom per year. [file 12889_2021_10671_MOESM1_ESM.docx]

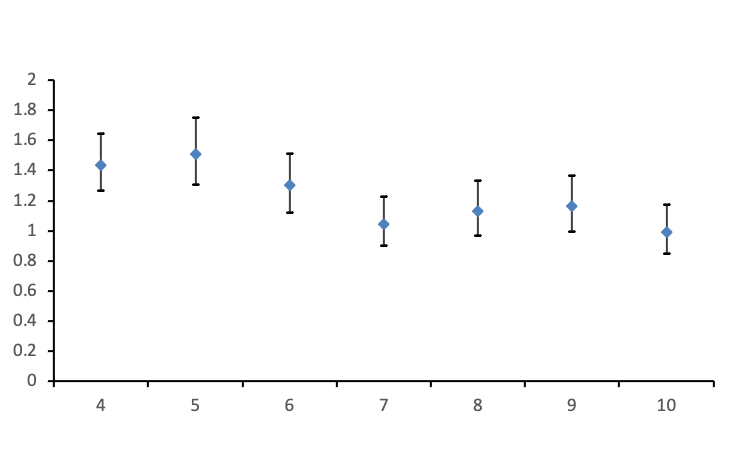


Supplement Figure 1. Relative risks of allergic rhinitis associated with daily mean temperature at 75th percentile relative to median temperature at lag02 day using different degrees of freedom per year.
